# Early detection of students’ mental health issues from a traditional daily health observation scheme in Japanese schools and its digitalization

**DOI:** 10.3389/fpubh.2024.1430011

**Published:** 2024-09-09

**Authors:** Tomoko Nishimura, Manabu Wakuta, Yuko Osuka, Nobuaki Tsukui, Ikue Hirata, Michio Takahashi, Masaki Adachi, Taiichi Katayama, Kyoko Aizaki, Motofumi Sumiya, Sayaka Kawakami, Toshiki Iwabuchi, Atsushi Senju

**Affiliations:** ^1^Research Center for Child Mental Development, Hamamatsu University School of Medicine, Hamamatsu, Japan; ^2^United Graduate School of Child Development, Osaka University, Suita, Japan; ^3^Institute of Child Developmental Science Research, Hamamatsu, Japan; ^4^Smart-Aging Research Center, Tohoku University, Sendai, Japan; ^5^Department of Psychology, Meiji Gakuin University, Tokyo, Japan

**Keywords:** mental health, school-based screening, daily health observation, digitalization, school absenteeism, depression, anxiety

## Abstract

**Objective:**

The implementation of school-based mental health screening offers promise for early detection of mental health issues in children; however, various barriers hinder its widespread adoption. This study aimed to investigate the predictive value of digital data obtained from an established daily health observation scheme in Japanese schools to identify later mental health issues in children.

**Methods:**

Data for the analysis were obtained from 2,433 students enrolled in five public schools. The data acquisition period spanned 76 school days, from September 1, 2022, to December 23, 2022, and student absences were recorded during this period. Depressive and anxiety symptoms were assessed in January 2023. The students’ daily physical and emotional health status was recorded as “daily health issue” scores and group-based trajectory modeling was employed to classify the long-term trends in these scores. Additionally, rolling z-scores were utilized to capture variability in daily health issue scores, with z-scores above +1 considered unusual responses.

**Results:**

After 4 months of daily health observations, students’ response trends were classified into five trajectory groups. The group experiencing the highest number of daily health issues (Group 5; 5.4% of the sample) exhibited more subsequent depressive and anxiety symptoms compared to the group with fewer issues (Group 1; 47.5%) (incident rate ratio [IRR] = 5.17; 95% confidence interval [CI]: 3.82, 6.99). Group 5 also demonstrated significantly more days of absence than Group 1 (IRR = 2.14, 95% CI: 1.19, 3.85). The average daily health issue scores for the entire period were associated with both depressive/anxiety symptoms and the number of days absent from school (IRR = 1.59, 95% CI: 1.45, 1.73; IRR = 1.18, 95% CI: 1.04, 1.35, respectively). Furthermore, a higher number of unusual responses during the entire period was also associated with more depressive/anxiety symptoms (IRR = 1.10, 95% CI: 1.07, 1.12).

**Conclusion:**

The current study is the first to demonstrate the predictive capability of a traditional daily health observation scheme to identify mental health issues in children. This study highlights the scheme’s potential to screen and safeguard children’s mental health, emphasizing the importance of digitalization and collaboration with various stakeholders.

## 1 Introduction

One of the fundamental goals of child psychology and psychiatry is to safeguard the mental health and well-being of children. However, research has established that the prevalence of mental disorders is higher among young individuals compared to those in other stages of life (1, 2), with nearly one in seven young people meeting the diagnostic criteria for a mental health disorder (3) and two in five scoring above the thresholds for emotional problems, conduct problems, or hyperactivity (4). The economic repercussions of mental health problems include direct costs of treatment and indirect social costs, such as reduced economic productivity and increased rates of unemployment (5). In 2010, mental health problems were estimated to have cost the global economy approximately US$ 2.5 trillion (US$ 1.7 trillion in social costs and US$0.8 trillion in direct costs) (6). Despite an annual expenditure of about US$ 3.7 billion on mental health research worldwide, there remains a need for further research in areas such as detection, screening, and diagnosis, which currently receive only 5% of relevant research funding, with the majority allocated to basic research like etiology (7).

To better understand and safeguard children’s development and mental health, it is imperative to conduct research within schools. Given that children spend a significant amount of time in school environments, schools play a crucial role in shaping various aspects of child development, including peer relationships, social interactions, academic achievement, cognitive progress, emotional control, behavioral expectations, and physical and moral growth, all of which influences, as well as are influenced by, children’s mental health (8). While maintaining stable mental health can positively impact a child’s overall development, challenges in social interactions and academic performance can exacerbate mental health issues, potentially leading to serious consequences such as school attendance problems (9, 10).

School absences have risen following the COVID-19 pandemic compared with pre-pandemic levels. The rate of persistent absenteeism (defined as missing >10% of academic sessions) in the USA and England has been reported to be approximately double the pre-pandemic rate (11, 12). COVID-19 has been reported to affect mental health and wellbeing, especially in children and adolescents (13), and may also be associated with increased absenteeism. Japan is no exception to this. In Japan, “*Futoukou*” (school non-attendance or school refusal) is defined as absence from school for 30 days (about 15% of academic sessions) or more per year due to some psychological, emotional, physical or social factor or background that prevents or discourages school attendance (excluding those due to illness or financial reasons) (14). The number of “*Futoukou*” after the pandemic (2022) is approximately 65% higher than the pre-pandemic (2019) (15). In Japan, children whose academic and/or interpersonal needs are ignored by teachers, especially in a rigid system that overemphasizes uniformity and pressure to perform well on curriculum-based examinations, have been prone to absenteeism (16, 17). Children who were absent from school without a clear reason tended to be regarded as having some problem, either within themselves or their families (18). Physical symptoms and depressive/anxiety symptoms are found at very high rates among such children (19). There are many reports on the association between school attendance problems and physical and mental health issues, not only in Japan (20, 21).

Schools play a crucial role in the identification and screening of physical and mental health issues in children. Multiple reports have highlighted the importance of school-based screening for the early detection of mental health issues in children and adolescents (8, 22–24). Implementing mental health screening in schools can facilitate early diagnosis and treatment of childhood mental health concerns. This proactive approach to diagnosis and treatment can significantly enhance the quality of life throughout a child’s lifespan and reduce the current costs associated with addressing mental health issues in adolescence and adulthood (25). For example, a global cost–benefit analysis found that the impact of failing to address the mental health and psychosocial support needs of 10–17-year-old children and adolescents affected by humanitarian emergencies would result in the equivalent of a global US$203 billion loss of potential lifetime earnings (26). In addition, school-based support programs effectively address barriers to accessing treatment and positively impact school outcomes for youths (27).

Promising as it is, implementing school-based mental health screening proves challenging due to inadequate funding, inadequate staff training and supervision, and difficulty coordinating a full continuum of prevention and intervention services (28). Soneson et al. (29) identified time, resource, and cost constraints as the primary barriers to the feasibility of school-based screening initiatives. Moreover, a lack of training in mental health assessment and ‘role conflict’ were also cited as significant obstacles, i.e., conflicting opinions among teachers regarding their role in assessing mental health in schools, with some acknowledging their roles while others viewing it as beyond their core responsibilities (30). These challenges make it difficult to implement and maintain systems for monitoring children’s mental health and may have a detrimental impact on their mental health.

In addition to the logistical challenges described above, the target age for screening poses another major challenge. Routine screening for depression within primary care settings is recommended for adolescents aged 12–18 years (31). Despite reports indicating a 3% prevalence of major depressive disorder in younger children (aged 8–15 years) (32), few studies have examined the utility of screening for this age group (33). Notably, depressive and anxiety symptoms frequently manifest alongside physical symptoms (34). Medically unexplained physical symptoms, known as functional somatic symptoms, are consistently associated with depressive and anxiety symptoms and disorders during childhood and adolescence (35). Furthermore, a study investigating the joint trajectories of mental and somatic symptoms revealed that depression, anxiety, and somatic symptoms (such as headache, nausea, and stomach problems) coexist from adolescence through midlife (36). Therefore, focusing on inquiring about co-occurring physical symptoms, rather than depressive and anxiety symptoms, might be more feasible, particularly for younger children, and may serve as predictive indicators of mental health problems.

To address the challenges associated with school-based screening for mental health issues, we exploited the “daily health observation” scheme, a longstanding practice in Japanese schools. In Japan, the School Health and Safety Act mandates that teachers and school staff conduct health observations, with the objectives of (1) detecting and intervening in children’s mental and physical health problems at early stages, (2) identifying outbreaks of infectious diseases and food poisoning and preventing their spread, and (3) promoting children’s interest in health management and self-care through continuous daily implementation (37). Traditionally, daily health observation data have been extensively used by teachers in practice (38, 39); however, they remain rarely used for research purposes despite the vast amount of accumulated data, partly because they were predominantly recorded on paper, limiting their accessibility to researchers. However, the situation is changing with the rapid digital transformation (Dx) occurring in Japanese schools. Japan’s “GIGA (global and innovation gateway for all) School Program” was accelerated during the COVID-19 pandemic, providing one tablet computer per student to ensure equitable and individually optimized learning (40). By July 2021, approximately 96% of elementary and junior high schools had begun using computers (41). Seizing this unique opportunity, we developed an app for daily health observation and an online system to digitally collate and store the obtained daily health data. This system not only enables longitudinal data analysis but also facilitates traditional daily health checks by teachers.

Our study aligns with the growing popularity of daily life studies, which typically measure constructs multiple times per day for extended periods. The increasing popularity of such studies can be attributed to the widespread use of portable electronic devices (42). Target constructs include mental and physical problems such as affect, mood/anxiety, and sleep using experience sampling methodology (ESM; an intensive longitudinal data collection technique) or various types of sensors (such as accelerometers) (42, 43). While most of these studies have been conducted with adults, research involving children and adolescents in school settings has delved into students’ social interactions and related experiences (44, 45). However, data collection was limited to a fixed survey period (often 7 days) (44) and conducted with relatively small samples. To our knowledge, no study has systematically collected data from schools over the course of months and utilized them for mental health screening.

This study aimed to explore the possibility of predicting mental health problems using technology that digitizes and longitudinally aggregates data from daily health observations. Specifically, we investigated whether both perspectives to capture long-term trajectories of daily health issues and short-term day-to-day variations could predict subsequent depressive and anxiety symptoms, as well as school absenteeism.

## 2 Methods

### 2.1 Participants

Data for the analysis were obtained from 2,433 participants (1,330 elementary school students [1st–6th grades] and 1,103 junior high school students [7th–9th grades]; 49.4% female) enrolled in five public schools (comprising three elementary and two junior high schools) situated in a city in western Japan. The city has a population of approximately 380,000 and contains 54 elementary and junior high schools. It is a medium-sized city with many communal houses adjacent to a large city. Eligible schools were selected by the Local Board of Education, and participation was contingent upon the agreement of each school’s principal. Data were obtained during daily life at the school and were used secondarily in this study. The data acquisition period spanned 76 school days, from September 1, 2022, to December 23, 2022.

### 2.2 Ethical considerations

All surveys were conducted during the school day. Parents were informed of the survey through a document from the school and provided consent for the secondary use of the school data in an opt-out format. Students were informed about the survey through explanations provided by their teachers, and no refusal for participation was offered. The study was conducted in accordance with the Code of Ethics of the World Medical Association (Declaration of Helsinki) and was approved by the Ethics Committees of Hamamatsu University School of Medicine (Refs 20-036; 22-230).

### 2.3 Measurements

**Figure 1:**
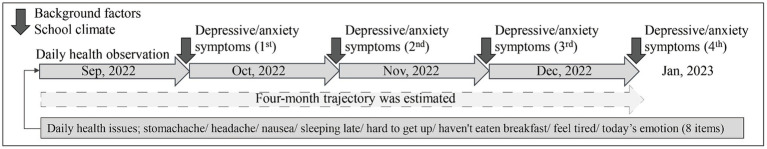
Data acquisition schedule and measurement items.

The data acquisition schedule and measurement items are shown in Figure 1.

#### 2.3.1 Daily health observation

Students logged their daily health status using tablet computers provided at school. The items related to physical health and activities of daily living were as follows: “I have a stomachache,” “I have a headache,” “I feel nauseated,” “I went to bed late last night,” “I had difficulty getting up in this morning,” “I skipped breakfast,” “I feel tired,” “I have an injury,” “I have a cold,” and “I feel unwell in another way.” These items were determined through discussions in a focus group consisting of teachers from the participating Local Board of Education, pedagogy and child development specialists, and school counselors. Items on the rhythm of life were added to the traditional health observation items. Emotional items were selected with reference to Zones of Regulation (46), which provides an easy way to think and talk about how we feel on the inside and sorts emotions into four colored zones, and students were prompted to select one out of the following four options: (1) happy, calm, energetic, motivated; (2) tired, sleepy, anxious, sad; (3) irritable, tense, restless, complaints; and (4) frustrated, angry, excited, and panicked. Any choice other than the first option (e.g., happy) was classified as an emotional issue.

In a preliminary analysis, an exploratory factor analysis was conducted using data collected 1 week after the start of the measurement period. A one-factor solution was selected based on eigenvalues (the first three eigenvalues were 1.99, 0.43, 0.13). Out of the 11 items mentioned above, the total scores for eight items were utilized as the “daily health issue” scores, excluding three items (injuries, colds, and unwell in another way) with low factor loadings. The reliability coefficient (Cronbach’s alpha) for this one-factor structure was calculated as 0.71.

Additionally, daily health observations included an item regarding school attendance. If a student was absent, the homeroom teacher recorded the absence. Consequently, the total number of days of absence during the survey period was tallied. However, detailed reasons for the absences were not recorded.

#### 2.3.2 Depressive/anxiety symptoms

Data were collected in January 2023 using the Patient Health Questionnaire-4 (PHQ-4) (47, 48). This scale comprises four items, with two extracted from the PHQ-9 (49) and two from the Generalized Anxiety Disorder-7 (GAD-7) (50). Notably, the item from the PHQ-2, “Feeling down, depressed or hopeless,” was modified to “Feeling down, depressed, irritable, or hopeless,” aligning with an item developed to evaluate depressive symptoms in children and adolescents in previous studies (51, 52). We examined whether the PHQ-4 should be treated as one factor or two factors by confirmatory factor analysis and reliability/validity testing. We concluded that it is reasonable to treat it as one factor (Supplementary Tables S1, S2). Each item was rated on a four-point Likert scale, and the total score was calculated. Higher scores indicate greater severity of depressive and anxiety symptoms.

#### 2.3.3 Student background factors

Students’ grades, gender, and primary language spoken (Japanese or non-Japanese) were collected.

#### 2.3.4 School environmental factors

Students’ perceptions of school climate were measured using the Japan School Climate Scale (JaSC) (53) in July 2022, prior to the health observation survey. The JaSC comprises 32 items designed to inquire how students feel about safety, learning, and interpersonal relationships within the school setting. The mean score of the 32 items was calculated for each student, while the average score was calculated for each classroom.

### 2.4 Statistical analysis

All statistical analyses were performed using Stata 17.0. The daily health issue scores (i.e., total scores of the eight items) obtained during the 4-months from September 2022 to December 2022 were treated as longitudinal data. These scores were classified into trajectory groups using group-based trajectory modeling (54). This modeling technique, a specialized application of finite mixture modeling, is designed to identify clusters of individuals exhibiting similar outcome progressions over time. We estimated this model by installing the “*traj*” command in Stata (55). Since the scores were not normally distributed, a zero-inflated Poisson (zip) model was employed. To determine the optimal number of groups, various models with 2–6 distinct trajectory groups were tested. Model fit selection was based on criteria such as a lower value of Bayesian Information Criterion (BIC) or sample size adjusted BIC (SA-BIC), a posterior-probability of group membership >0.7, and a sufficient sample size of ideally ≥5% in each group (56, 57).

Associations between depressive/anxiety symptoms, the number of days absent from school, and the identified trajectory groups were each explored using multilevel negative binomial regression models. The models incorporated sex, grade, and language as student-level covariates, and the classroom average of the school climate scores as a classroom level covariate.

One limitation of the trajectory group analysis was its inability to capture day-to-day variations in daily health issue scores, as it represents the average response trends of students. We derive a rolling z-score, which is calculated from the 7-day rolling average and standard deviation (SD) to capture such day-to-day variations. The rolling z-score is a commonly used metric for visualizing trends and detecting anomalies (58). We defined a z-score > +1 as an unusual response and the total count of such scores during the study period was tallied. Similar multilevel negative binomial regression models were then employed to investigate associations between the average daily health issue scores, total number of unusual responses, depressive/anxiety symptoms, and the number of days absent from school. Associations for each month over the entire study period were examined in the full sample.

## 3 Results

Among the 2,433 participants, 290 (11.9%) were excluded from the analysis due to low response rates (less than half of the total). Of these, 38 students did not respond at all. The remaining 2,143 students (1,241 elementary and 902 junior high school students) were included in the analysis (Table 1). The number of responses ranged from 39 to 76 (mean = 63.9; SD = 8.5).

**Table 1:** Demographic characteristics of the total sample.

Participants	Total (*n* = 2,143)
Female sex; *n* (%)	1,059 (49.4%)
Elementary school student; *n* (%)	1,241 (57.9%)
Junior high school student; *n* (%)	902 (42.1%)
Non-Japanese; *n* (%)	105 (4.9%)
Depressive/anxiety symptoms in January 2023; median (IQR)	1 (0–3)
Four-month total number of days absent from school; median (IQR)	0 (0–1)

IQR, inter quartile range.

Table 2 shows the model fit indices for group-based trajectory modeling. While the absolute values of the BIC and SA-BIC decreased with an increase in the number of groups, convergence was not observed for the 6-group solution. The entropy remained consistently high (> 0.90) for all five solutions. The percentage of members in each group was at least 5% in the 5-group solution, which was deemed the optimal solution based on these results.

**Table 2:** Model fit indices.

Number of groups	BIC	SA-BIC	Entropy	Proportion
2	−110072.27	−110059.80	0.980	72.3%; 27.7%
3	−100764.92	−100783.63	0.972	60.5%; 26.6%; 12.9%
4	−97819.05	−97794.11	0.929	54.5%; 20.7%; 17.3%; 7.5%
5	−95746.63	−95713.37	0.900	47.4%; 21.8%; 14.1%; 11.3%; 5.4%
6	No convergence			

BIC, Bayesian Information Criterion; SA-BIC, sample size adjusted BIC.

**Figure 2:**
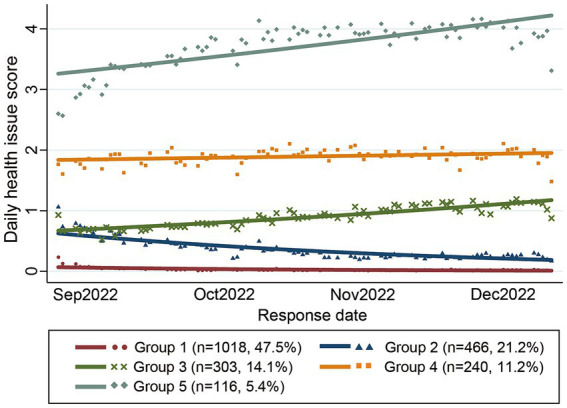
Trajectories of the daily health issue scores in each identified group.

The trajectories of the five groups are depicted in Figure 2. Group 1 was characterized by almost no health issues throughout the study period, comprising the largest proportion of students (47.5%). Group 2 experienced less than one daily health issue, with a declining trajectory in the latter half of the study; 21.2% of students were assigned to this group. Group 3 displayed less than one daily health issue during the first half of the period, which gradually increased toward the second half of the study period, with 14.1% of students assigned to this group. Group 4 exhibited approximately two daily health issues, representing 11.2% of the students. Group 5 had the most daily health issues (3–4 issues), which increased toward the second half of the period; 5.4% of students were assigned to this group.

The demographic characteristics of the five groups and their differences are presented in Table 3. Differences among the groups were observed in terms of gender, grade, depressive/anxiety symptoms across all periods, four-month average daily health issue scores, and four-month total number of unusual responses. While most students had zero school absences, variations were noted between the groups for the entire study period and in December.

**Table 3:** Demographic characteristics of each trajectory group.

	Group 1 (*n* = 1,018, 47.5%)	Group 2 (*n* = 466, 21.2%)	Group 3 (*n* = 303, 14.1%)	Group 4 (*n* = 240, 11.2%)	Group 5 (*n* = 116, 5.4%)	Group differences
Female sex; *n* (%)	525 (51.7%)	228 (49.0%)	131 (43.5%)	126 (53.9%)	49 (42.6%)	^a^*χ^2^* (4) = 10.2, *p* = 0.04
Grade 1; *n* (%)	76 (7.5%)	62 (13.3%)	33 (10.9%)	38 (15.8%)	21 (18.1%)	^a^*χ^2^* (32) = 157.1, *p* < 0.001
2; *n* (%)	114 (11.2%)	58 (12.5%)	34 (11.2%)	23 (9.6%)	11 (9.5%)	
3; *n* (%)	88 (8.6%)	64 (13.7%)	33 (10.9%)	22 (9.2%)	7 (6.0%)	
4; *n* (%)	69 (6.8%)	56 (12.0%)	34 (11.2%)	21 (8.8%)	14 (12.1%)	
5; *n* (%)	74 (7.3%)	37 (7.9%)	37 (12.2%)	27 (11.3%)	15 (12.9%)	
6; *n* (%)	64 (6.3%)	43 (9.2%)	33 (10.9%)	18 (7.5%)	15 (12.9%)	
7; *n* (%)	191 (18.8%)	68 (14.6%)	30 (9.9%)	46 (19.2%)	12 (10.3%)	
8; *n* (%)	157 (15.4%)	51 (10.9%)	45 (14.9%)	33 (13.8%)	12 (10.3%)	
9; *n* (%)	185 (18.2%)	27 (5.8%)	24 (7.9%)	12 (5.0%)	9 (7.8%)	
Junior high school student; *n* (%)	146 (31.3%)	533 (52.4%)	99 (32.7%)	91 (37.9%)	33 (28.5%)	^a^*χ^2^* (4) = 87.8, *p* < 0.001
Non-Japanese; n (%)	42 (4.1%)	32 (6.9%)	12 (4.0%)	12 (5.0%)	7 (6.0%)	^a^*χ^2^* (4) = 6.1, *p* = 0.19
Depressive/anxiety symptoms in January 2023; median (IQR)	0 (0–2)	1 (0–3)	2 (0–4)	2 (0.5–4.5)	4 (2–6)	^b^*χ^2^* (4) = 265.4, *p* < 0.001
Four-month total number of days absent from school; median (IQR)	0 (0–1)	0 (0–1)	0 (0–1)	0 (0–1)	0 (0–1)	^b^*χ^2^* (4) = 13.2, *p* = 0.01

a χ–square test.

b Kruskal–Wallis equality-of-populations rank test.

IQR, inter quartile range.

Table 4 illustrates the results of multinomial negative binomial regression testing for group differences in depressive/anxiety symptoms and the number of days absent. Using Group 1 as the reference, all other groups had higher depressive/anxiety symptoms, with Group 5 exhibiting the highest incident rate ratio (IRR) of 5.17 (95% confidence interval [CI]: 3.82, 6.99) compared to Group 1. Group 5 also demonstrated significantly more days absent than Group 1 (IRR = 2.14, 95% CI: 1.19, 3.85). Conversely, there were no significant differences in the number of days absent among the other groups compared to Group 1.

**Table 4:** Association between depressive/anxiety symptoms, four-month total number of days absent from school, and health issue trajectory groups.

	Depressive/anxiety symptoms (January 2023)		Total number of days absent from school	
	IRR	95% CI	IRR	95% CI
Student level				
Group 1	Reference		Reference	
Group 2	1.96^**^	1.54, 2.48	1.00	0.69, 1.45
Group 3	2.50^**^	1.97, 3.16	1.09	0.68, 1.74
Group 4	3.23^**^	2.55, 4.10	1.29	0.84, 1.98
Group 5	5.17^**^	3.82, 6.99	2.14^*^	1.19, 3.85
Grade	0.99	0.93, 1.06	0.90	0.59, 1.37
Female sex	1.15	0.99, 1.33	0.86	0.69, 1.07
Language	1.00	0.70, 1.42	1.22	0.54, 2.78
Classroom level				
School climate	0.58^*^	0.36, 0.93	13.26	0.68, 259.7

IRR, incident rate ratio; 95% CI, 95% confidence interval. ^*^ *p* < 0.05, ^**^ *p* < 0.001.

In its upper section, Table 5 presents the associations between the average daily health issue scores, depressive/anxiety symptoms, and the number of days absent for each month and the entire period. Daily health issue scores were associated with depressive/anxiety symptoms across all periods and with the number of days absent during the latter half and the entire period.

**Table 5:** Association between daily health issue scores (upper section), or total number of unusual responses (lower section), depressive/anxiety symptoms, and number of days absent from school in each month and for the entire period in the full sample.

	Depressive/anxiety symptoms			Number of days absent from school		
	Measurement time	IRR	95% CI	Measurement period	IRR	95% CI
Average daily health issue scores						
Sep. 2022	1st (Oct. 2022)	1.58^**^	1.45, 1.68	Sep. 2022	1.05	0.83, 1.33
Oct. 2022	2nd (Nov. 2022)	1.42^**^	1.31, 1.53	Oct. 2022	1.03	0.80, 1.36
Nov. 2022	3rd (Dec. 2022)	1.44^**^	1.34, 1.55	Nov. 2022	1.21^*^	1.03, 1.42
Dec. 2022	4th (Jan. 2023)	1.43^**^	1.33, 1.54	Dec. 2022	1.28^*^	1.06, 1.54
Entire period	4th (Jan. 2023)	1.59^**^	1.45, 1.73	4 months	1.18^*^	1.04, 1.35
Total number of unusual responses						
Sep. 2022	1st (Oct. 2022)	1.23^**^	1.17, 1.29	Sep. 2022	0.89	0.76, 1.04
Oct. 2022	2nd (Nov. 2022)	1.27^**^	1.20, 1.35	Oct. 2022	0.88	0.70, 1.09
Nov. 2022	3rd (Dec. 2022)	1.24^**^	1.17, 1.32	Nov. 2022	0.97	0.83, 1.14
Dec. 2022	4th (Jan. 2023)	1.23^**^	1.14, 1.33	Dec. 2022	1.01	0.82, 1.25
Entire period	4th (Jan. 2023)	1.10^**^	1.07, 1.12	4 months	1.02	0.98, 1.05

IRR, incident rate ratio; 95% CI, 95% confidence interval. ^*^ *p* < 0.05, ^**^ *p* < 0.001.

In the lower section of Table 5, associations between the total number of unusual responses (rolling z-score > +1), depressive/anxiety symptoms, and the number of days absent are delineated for each month and the entire period. The total number of unusual responses was associated with depressive/anxiety symptoms across all periods but not with the number of days absent from school.

## 4 Discussion

The current study demonstrated, for the first time, that it is possible to predict later depressive and anxiety symptoms using data from daily physical and emotional health observations conducted within the framework of the daily health observation scheme long incorporated into schools in Japan. This finding suggests that school-based mental health screening is feasible when data from daily health observations can be used through digitization. Leveraging such systems could help overcome some of the reported logistical barriers (such as time, resources, and cost issues) that may arise when introducing and continuing a new mental health screening system in schools. Note that the current study was made possible through a unique combination of the traditional system of daily health observation, the new system of digitization, and collaboration with educational professionals, offering promising avenues for implementing a new system aimed at screening and safeguarding children’s mental health.

Three indices were derived in this study. Firstly, individual “daily health issue” scores were calculated as a measure of daily physical and emotional health. Secondly, the children were categorized into a trajectory group of long-term health status, estimated based on their daily health issue scores. Lastly, a rolling z-score was calculated to identify the level of variability in each child’s scores.

The “daily health issue” score, a total score of eight items related to physical and emotional health, predicted later depressive and anxiety symptoms. These items include physical symptoms such as stomachache, headache, and fatigue, which have been shown to be associated with depression and anxiety. A meta-analysis by Henningsen et al. (59) reported that chronic fatigue syndrome is particularly associated with depression. In addition, sleep problems, which were included as “daily health issue” items, were reported to predict higher levels of depression and depression/anxiety symptoms (60). Relevant mechanisms, especially in adolescence, include biological mechanisms such as alterations in corticolimbic and mesolimbic brain circuits, psychological mechanisms such as cognitive inflexibility and interpretational biases, social mechanisms such as reduced social interactions and family disfunction, and interactions of these mechanisms (61). The “daily health issue” score was also associated with the number of days absent from school. Since a large body of literature also suggests that physical symptoms, sleep, and emotional problems are associated with school attendance problems (10, 12), the daily accumulation of scores consisting of such items may contribute to the prediction and early detection of school attendance problems.

Five trajectory groups were identified after examining the long-term trajectories of daily health issue scores. Approximately half of the students were assigned to a trajectory group with a score of 0 almost every day; however, approximately 5% scored 3 or higher every day. Because this trajectory group with high scores was shown to have particularly high depressive and anxiety symptoms and high absenteeism from school, immediate intervention is recommended to prevent the deterioration of mental health and school refusal. However, the 5% rate is slightly low compared to the reported prevalence rates of depression and anxiety disorders: Prevalence rates were reported to be 1.7% for depression and 6.6% for anxiety disorders among children aged 6–11 and 6.1% for depression and 10.5% for anxiety disorders among those age 12–17 (62). This lower rate might partly be because not all students with depressive/anxiety symptoms reported multiple daily health issues sufficient to be categorized into the risk group, which would be missed in analyses that only focused on one-day scores or long-term trends. Based on these findings, we analyzed the tendency for an unstable condition or the magnitude of score variability quantified with rolling z-scores to further identify high-risk children.

Rolling z-scores were used to detect high variability in the daily health scores, with scores surpassing the average response of the preceding 7 days flagged as “unusual responses.” The higher the number of unusual responses, i.e., the greater the variation in scores, the higher the depressive and anxiety symptoms. Group 5, which had the highest daily health issue scores, exhibited the most unusual responses. Interestingly, Group 1, which had the lowest daily scores, also displayed unusual responses, which might have been overlooked in the daily score or trajectory analysis. Given that these unusual responses are more sensitive to transient changes in children’s responses than long-term trajectories, it could lead to prompt support for students, potentially mitigating the deterioration of their mental health. Importantly, we found that a simple formula, the rolling z-score, could be used to detect daily variations and potentially be used to predict mental health problems. Its simplicity as an algorithm has the advantage of allowing teachers to explain why variation was detected. We need to use these scores and tools as an aid to understanding and supporting students without becoming overly dependent on the algorithms (63).

Daily health observation data can help predict mental health deterioration and absenteeism, as well as improve students’ mental health literacy. Defined as the understanding of acquiring and maintaining positive mental health, comprehending mental disorders and their treatments, reducing associated stigma, and enhancing help-seeking efficacy (i.e., knowing when and where to seek help and developing competencies designed to improve mental health care and self-management capabilities), mental health literacy plays a crucial role (64, 65). Leveraging health observation data can empower students to comprehend their health status and effectively manage deteriorating health conditions.

This study did not analyze by grade level (elementary and junior high school), but several differences were found between grade levels. First, the assignment of the five trajectory classes differed by grade level (Table 2). Junior high school students tended to be classified more frequently in Class 2, where they reported relatively fewer health issues. In contrast, elementary school students tended to be classified in the other classes. Second, with regard to depressive and anxiety symptoms, 11.4% of elementary school students and 7.0% of junior high school students were classified as having moderate or severe symptoms, a larger percentage of elementary school students (*χ^2^* (1)=10.6, *p* = 0.001). According to a report from Japan, about 10–15% of 4th–6th graders in elementary school and 12–16% of 7th–9th graders in junior high school had moderate or severe depression (51). Previous studies have also shown that older children tend to have higher rates of depressive and anxiety symptoms (62), further validation of the grade-level differences is needed by expanding the target population. Third, the percentage of students who were absent for 10% or more of the total period during the observation period was higher among middle school students (*χ^2^* (1) = 5.37, *p* = 0.02), with 2.5% of elementary school students and 4.2% of middle school students, which is similar to previous reports (66). Although the factors contributing to absenteeism were not clear in this study, it has been reported that factors may vary by age, such as separation anxiety disorder being more common among younger children, and social phobia being more common in older children and adolescents (67). Early prevention and intervention of persistent absenteeism is important, considering age-related differences in factors contributing to absenteeism.

While this study clearly demonstrates the feasibility of predicting mental health status using daily health observation data from a relatively large sample, it has some limitations. Firstly, the absence of data input for students who are not present in school poses a significant challenge because daily health observations are conducted in school settings. It is worth mentioning that students excluded from our analysis were those frequently absent (number of absences from school in included students: mean = 0.91, SD = 2.12; excluded students: mean = 6.75, SD = 17.21; *χ^2^* (1) = 10.2, *p* = 0.001). The lack of data from these frequently absent students poses a major challenge for future mental health screening at schools because monitoring physical and emotional health is even more important for students who are frequently absent, many of whom could be at higher risk for mental health problems. Thus, enabling data input even during absences is imperative. Despite revealing a partial link between health observation data and absenteeism, this association might have been underestimated due to the exclusion of frequently absent students from our analysis. We posit that the prediction of school absenteeism can be further improved by obtaining health observation data on the day of absence. Secondly, the reason for the absence was not identified. Absenteeism refers to excusable absences related to medical illness or injury, or inexcusable absences related to environmental, social, psychiatric, or other conditions (68). In addition, inexcusable absences may have a variety of causes, including school withdrawal, school refusal behavior, and truancy. This study could not address the heterogeneity in absenteeism and relevant mental health issues. If it were possible to distinguish the types of absences and address the heterogeneity, more accurate predictions would be possible. Thirdly, approximately 15.9% of the data utilized in our analysis were missing, attributed to system errors (e.g., network problems) and, more importantly, non-responders. This raises concerns regarding the potential underreporting of physical and emotional health concerns by some students, thereby hindering the school’s ability to address their needs. Thus, it is important to develop strategies to address non-responses and ensure inclusivity while respecting students’ right not to report their own condition. Fourth, physical symptoms were used to predict mental health status; however, it remains unclear whether these symptoms are solely related to mental health. It is meaningful to ask about physical symptoms to detect signs of deteriorating mental health, given that young children may not be fully aware of their mental states, which often manifest as physical symptoms instead (34, 69). Nonetheless, it is also important not to miss the deteriorating physical health. Lastly, this study was based on an existing daily health observation scheme unique to Japan, limiting the generalizability of its findings to other countries lacking similar systems. However, the widespread use of digital devices in school environments, primarily for educational purposes, is becoming prevalent in many countries (70), offering opportunities for these schools to leverage digital platforms for health monitoring. We hypothesized that such school-based brief daily health observations would help screen for mental health problems in other countries once implemented; however, future research is warranted to test our hypothesis.

Future endeavors in utilizing daily health observation data should delve into strategies to provide intervention support to students who are at risk of mental health deterioration and school absenteeism. Assessment with high specificity and potential is also needed to clearly identify students’ mental health needs and provide more prescriptive intervention recommendations. Werner-Seidler et al. (71) highlighted that targeted prevention programs tailored for young people with risk factors or symptoms have greater effect sizes than universal depression prevention programs. Furthermore, initiatives like the Interconnected Systems Framework (ISF) aim to systematically integrate positive behavioral interventions and support (PBIS) with school-based mental health promotion, prevention, and intervention, showing promising effectiveness (72).

In conclusion, the current study demonstrated the potential of daily health observation data to predict mental health deterioration in school-aged children, focusing on daily health scores, day-to-day variations, and long-term trends. Early identification and intervention of mental health problems in children are important as they often have a negative effect on children’s everyday functioning and wellbeing, as well as long lasting effects later in life (73). Establishing a detection and screening system based on these findings would lay a foundation for developing targeted interventions for at-risk students and integrating daily health observations into preventive mental health literacy education.

## Data Availability

The data analyzed in this study is subject to the following licenses/restrictions: the data are not publicly available because they contain information that could compromise the privacy of research participants. Requests to access these datasets should be directed to Tomoko Nishimura, tomoko.n@hama-med.ac.jp.
