# Supplementary material for: Early detection of students’ mental health issues from a traditional daily health observation scheme in Japanese schools and its digitalization
Source: Front Public Health. 2024 Sep 9;12:1430011. doi: 10.3389/fpubh.2024.1430011 (PMC11416938; doi:10.3389/fpubh.2024.1430011)
Supplement: Supplementary file 1 [file Data_Sheet_1.PDF]

Supplementary Material

Supplementary Table 1. Model fit indices from 1-factor to 2-factor solutions for Patient Health Questionnaire (PHQ)

Factor solutions	1-factor	2-factor	Threshold[1]
Chi-square test p-value	<.001	<.001	>.05
CFI	.994	1.000	>.90
RMSEA	.065	.000	<.08
SRMR	.016	.002	<.08

Note: Model fit was tested using the comparative fit index (CFI), Tucker–Lewis index (TLI), the root-mean-square error of approximation (RMSEA), and Root Mean square Residual (SRMR).

1. Hu L, Bentler PM. Cutoff criteria for fit indexes in covariance structure analysis: Conventional criteria versus new alternatives. *Struct Equ Model* (1999) 6(1), 1–55. doi:10.1080/10705519909540118

Supplementary Table 2. Reliability and validity from 1-factor to 2-factor solutions for the Patient Health Questionnaire (PHQ)

1-factor			2-factor						
Depression/Anxiety			Depression			Anxiety			
α	CR	AVE	α	CR	AVE	α	CR	AVE	HTMT
.818	.820	.538	.627	.638	.474	.793	.795	.660	.930

Note: Reliability was evaluated using Cronbach's α with a criterion value of .70 or higher[2] and composite reliability (CR) with a criterion value of .70 or higher.[3] Convergent validity was evaluated using the average variance extracted (AVE), with a criterion value of .50 or higher.[4] Discriminant validity was evaluated using the heterotrait-monotrait ratio of correlations (HTMT), with a criterion value of .85 or lower.[5]

In the two-factor solution, reliability and convergent validity were not sufficiently high for the depression subscale. Discriminant validity did not meet criterion values for the two-factor solution. Based on these results, we conclude that it is appropriate to treat the PHQ as a single factor.

2. Hair JF, Black WC, Babin BJ. Multivariate data analysis: A global perspective (7th ed.), Pearson Education. (2010).
3. Bagozzi RP, Yi Y. On the evaluation of structural equation models. *JAMS* (1988) 16;74–94. doi:10.1007/BF02723327
4. Fornell C, Larcker DF. Evaluating structural equation models with unobservable variables and measurement error. *J Mark Res* (1981) 18(1); 39-50.
5. Henseler J, Ringle CM, Sarstedt M. A new criterion for assessing discriminant validity in variance-based structural equation modeling. J Acad Mark Sci (2015) 43;115–135. doi:10.1007/s11747-014-0403-8
